# Brief Developmental Exposure to Fluoxetine Causes Life-Long Alteration of the Brain Transcriptome in Zebrafish

**DOI:** 10.3389/fendo.2022.847322

**Published:** 2022-04-28

**Authors:** Amin Nozari, Remi Gagné, Chunyu Lu, Carole Yauk, Vance L. Trudeau

**Affiliations:** ^1^ Department of Biology, University of Ottawa, Ottawa, ON, Canada; ^2^ Environmental Health Science and Research Bureau, Health Canada, Ottawa, ON, Canada

**Keywords:** embryonic development, brain transcriptome, fluoxetine, life-long effect, RNA sequencing, zebrafish

## Abstract

Fluoxetine (FLX) and other selective serotonin reuptake inhibitors are widely used to treat depressive disorders during pregnancy. Early-life exposure to FLX is known to disrupt the normal function of the stress axis in humans, rodents, and teleosts. We used a zebrafish line with a cortisol-inducible fluorescent transgene to study the effects of developmental daily exposure to FLX (54 µg/L) on the transcriptomic profile of brain tissues in exposed larvae and later as 6-month-old adults. High throughput RNA sequencing was conducted on brain tissues in unstressed and stressed conditions. Long-lasting effects of FLX were observed in telencephalon (Tel) and hypothalamus (Hyp) of adult zebrafish with 1927 and 5055 genes significantly (≥1.2 fold-change, false-discovery p-value < 0.05) dysregulated in unstressed condition, respectively. Similar findings were observed in Hyp with 1245 and 723 genes being significantly dysregulated in stressed adults, respectively. Differentially expressed genes converted to *Homo sapiens* orthologues were used for Ingenuity Pathway Analysis. The results showed alteration of pathways involved in neuroendocrine signaling, cholesterol metabolism and synaptogenesis. Enriched networks included lipid metabolism, molecular transport, and nervous system development. Analysis of putative upstream transcription regulators showed potential dysregulation of *clocka* and *nr3c1* which control circadian rhythm, stress response, cholesterol metabolism and histone modifications. Several genes involved in epigenetic regulation were also affected by FLX, including *dnmt3a, adarb1, adarb2, hdac4, hdac5, hdac8*, and *atf2*. We report life-long disruptive effects of FLX on pathways associated with neuroendocrine signaling, stress response and the circadian rhythm, and all of which are implicated in the development of depressive disorders in humans. Our results raise concern for the persistent endocrine-disrupting potential of brief antidepressant exposure during embryonic development.

## Introduction

Selective serotonin reuptake inhibitors (SSRIs) are class C pharmaceuticals. This implies that they are likely to have adverse effects on the developing fetus. Despite this, SSRIs have been widely used to treat depression and mood disorders in pregnancy and early post parturition (1, 2). It is known that SSRIs cross the placental barrier during pregnancy (3, 4). In early development, serotonin has important roles in the development of neural circuits, particularly in cell division, cell differentiation, cell migration, myelination, and synaptogenesis (5–7). Therefore, dysregulation of serotonin production in the developing brain by SSRIs could have potential long-lasting effects on the neuronal networks in the fetal brain (8).

Human, rodent, and zebrafish studies show that developmental exposure to SSRIs could adversely affect social behaviour, neurodevelopment, vulnerability to stressful situations, and physiological homeostasis (9–11). Recently, teleosts have been used as amenable models to study the developmental effects of SSRIs (12–14). The serotonergic pathways are highly conserved between teleost and other mammals. For example, comparison of the amino acid residues with known function in SSRI binding to the serotonin reuptake transporter (*sert*, *slc6a4a*) indicates 93% identity between human and different fish species (15–17). Dose-dependent anxiolytic effects have been reported in fathead minnows (*Pimephales promelas*) upon exposure to sertraline and FLX (18, 19). Several serotonin (5HT) receptors have been implicated in modulating anxiety-like behaviours in fish, including 5-HT_1A_, 5-HT_1B_, 5-HT_1D_,5-HT_2A_, 5-HT_3_,5-HT_T5_, and 5-HT_T6_ (20–23). Moreover, teleost models have been used to discern the environmental impacts of SSRIs that reach aquatic systems through sewage treatment facilities (17, 24).

It has been suggested that altered regulation of the stress-axis mediates long-lasting effects of SSRIs on behaviour. For example, FLX exposure decreases circulating levels of glucocorticoids and downregulates glucocorticoid receptors (GR) in the nervous system in humans and rodents (25–29). These data are significant because downregulation of GR is linked to depressive-like behaviours in rodents (30). Impaired GR function in the central nervous system could result in hyperactivity of the HPA axis and behavioral abnormalities observed in depression (31). Studies in rodents show that stress contributes to serotonergic pathway dysregulation (31). A subpopulation of serotonergic neurons in raphe nuclei respond to an elevation of corticotropin-releasing hormone (CRH) and believed to be associated with anxiety-like behavior in rats (32). The close interaction between the serotonergic system and the stress-axis also occurs in teleosts. For example, high levels of the 5-HT_1A_ receptor are detected in preoptic area of the telencephalon (TEL), pituitary and interrenal cells of Gulf toadfish (*Opsanus beta*) (33). Activation of the 5-HT_1A_ receptor causes increases in *crh* levels and elevation of whole-body cortisol in Gulf toadfish and rainbow trout (*Oncorhynchus mykiss*) *(* 34). Moreover, subordinate fish have higher cortisol levels and serotonergic neural activity than dominant fish (15, 33, 35). Subordinate fish have an elevated mRNA levels for of 5-HT_1A_ and 5-HT_2A_ receptors in the TEL and increased levels of 5-HIAA (the primary metabolite of serotonin) specifically in the preoptic area (35).

Recent studies in our lab by Vera Chang et al. showed that developmental exposure to FLX results in hyporcortisolism in the descendant adults for more than three generations (14, 36, 37). Such decreases in whole-body cortisol levels coincide with a decrease in exploratory behavior of males in the novel tank test, an anxiety-like behavior in zebrafish (14, 36, 37). Global transcriptomic analysis of the interrenal revealed significant dysregulation of glucocorticoid signaling and circadian rhythm pathways in the FLX exposed lineage (F0 and F3) compared to unexposed controls zebrafish (14, 36, 37).

In the current study, using a transgenic zebrafish line, we studied the persistent effects of developmental exposure to FLX on genes involved in stress and neurogenesis in larvae and adult Tel and Hyp. Moreover, using pathways analysis, we evaluated the effect of such exposure on genes involved in the development of depression in human. We used the SR4G transgenic line, which expresses an enhanced green fluorescent protein (eGFP) when exposed to physical stress (38). We aimed to use the transgene transcript (GFP mRNA) as an internal control to confirm the stress response activation in our transcriptomic analysis. Since it has been suggested that the effect of FLX is sex-dependent in zebrafish and more prominent in males (14, 39), only male adults were studied in the current study. Our findings provide evidence for life-long effects of developmental exposure to FLX on pathways involved neural development, stress response, and cholesterol metabolism in adult males.

## Materials and Methods

### Animals and Husbandry

The SR4G transgenic zebrafish line was kindly provided by Dr. Karl Clark (Mayo Clinic, Minnesota, USA) and Dr. Xiao-Yan Wen (University of Toronto, Ontario, Canada) (38). One-day post-fertilization embryos were transferred to Ottawa and placed in the quarantine room of the University of Ottawa aquatic facility per regulations of Animal Care and Veterinary Services (ACVS). The embryos were allowed to hatch and were raised in the quarantine facility until they reached six months of age. These zebrafish were used to establish our founder generation. The F_0_ generation was derived from this founder line.

### Study Design

To study the life-long effects of developmental exposure to FLX on the central nervous system, we collected the larval heads, and brain tissues (Tel and Hyp) of male adult zebrafish. Two independent variables were studied: stress condition and FLX exposure (**Supplementary Figure 1**). A total of 12 treatment and control groups were studied. For the larval study, all experiments were conducted with six replicates (experimental units) which each consisted of 22-28 larval heads (sampling unit) from individuals that were raised in separate containers to avoid pseudo-replication (**Supplementary Figure 1**). Similarly, for adults, all experiments were conducted with six replicates (experimental units) prepared by pooling of 5 tissue samples (sampling unit: either Tel or Hyp) harvested from adult male zebrafish. The same adult was used to harvest the Tel and Hyp so that the pooled samples were from the same five adult zebrafish for Tel and Hyp in each replicate. The five individuals pooled together were raised in separate containers from other groups of 5 to avoid pseudo-replication (**Supplementary Figure 1**). The details about FLX exposure and stress routine is provided in the following sections.

### Chemical Exposure

The stock of 1 mg/ml of fluoxetine (FLX; CAS# 56296-78-7, Millipore-Sigma, Burlington, MA, USA) was prepared in 99% ethanol as the vehicle. To confirm concentrations in the working solution (54 µg/L), fluoxetine was quantified by comparing its LC/MS peak area with a serial dilution of the fluoxetine standard compound. The serial dilution was prepared in 0.1% formic acid, 20% acetonitrile in water (LC: Agilent 1100 capillary HPLC, Agilent, Santa Clara, CA, USA; MS: Thermo LTQ Linear Ion Trap mass spectrometer, Thermofisher, CA, USA). The HPLC gradient was set to 15% B (0.1% formic acid in acetonitrile) to 45% B (0.1% Formic acid in acetonitrile) over 15 mins. The mass spectrometer was set on MS1 scan mode between 150-1500 m/z range. Chromatograms were plotted by monoisotopic ion at 310.2 m/z. Embryos from on the day of fertilization (0 dpf) were exposed to freshly prepared 54 µg/L FLX daily until they reached 6 dpf. The control groups were exposed to the vehicle only (final concentration of 0.005% ethanol) from 0-6 dpf. The total water volume of the rearing tanks was adjusted to 1 ml per larva. The treatment and control larvae were further divided into unstressed and stressed groups and were housed in separate shelves in the incubator (PR505750L, Precision™ plant growth chamber, 504L; Thermofisher) to avoid unwanted stressors. The unhatched or dead embryos/larvae were removed once daily. After Day 6, control and FLX-exposed lineages were raised in clean water until they reached six months. At that time, the sexes were segregated, and subsequent experiments performed on male adult zebrafish. Both the control and FLX-lineages were further divided into unstressed and stressed groups.

### Net Handling Stress and Sampling for RNA Sequencing

For both FLX- treated larvae and adults and their corresponding controls, the unstressed groups were not exposed to any handling or mechanical stress and were sacrificed immediately on the start of light cycle on the day of an experiment using ice-cold water. The stressed groups were exposed to a modified handling net stress described previously (14). Briefly, the larvae from each group were exposed to air for 1 minute trapped in a mesh net, afterwards returned to the embryo medium for 3 minutes for rest, followed by another 1-minute exposure to air trapped in a mesh net (**Supplementary Figure 2**). The adults from each group were exposed to air for 3 minutes trapped in a mesh net, afterwards returned to the water for 3 minutes for rest, followed by another 3-minute exposure to air trapped in a mesh net. Sample collection was carried out 60 minutes after the last net stress based on our previous study (40). For 7 dpf larvae, a pooled sample of heads was acquired by removing the trunk and tail sections. This was done to reduce the number of abundant muscular and gastrointestinal-related genes in our data set. Each larval sample was a pool of 22-28 heads. The tissue samples were immediately frozen on dry ice and stored at -80 °C until further assessments. For adults, after careful dissection of the skull, the Tel and the Hyp were separately dissected and immediately placed in sample tubes and frozen on dry ice. Brain tissues from 5 adult fish were pooled and stored at -80°C until further experimentations. All pooled tissue samples were prepared in 6 replicates across all treatments (FLX-L: fluoxetine exposed larvae; FLX-Tel: Tel from adults exposed to FLX as larvae; FLX-Hyp: Hyp from adults exposed to FLX as larvae; cL: control larvae, cTel: control adult Tel; cHyp: control adult Hyp) (**Supplementary Figure 1**).

### Total RNA Extraction and Quality Control

Total RNA extraction from all samples was carried out using Qiagen RNeasy mini kits (Qiagen, Hilden, Germany) based on the manufacturer recommendations. The quality of the total extracted RNA was assessed using an Agilent 4150 TapeStation system (Agilent, Santa Clara, CA, USA). The RNA integrity number (RIN) was acquired for all samples and only those with RIN >8.5 were considered of sufficiently high quality and used for RNA sequencing (41).

### Next-Generation High Throughput RNA Sequencing

Next-generation high-throughput RNA sequencing was carried out using Illumina technology (Illumina, San Diego, CA, USA). A total of 72 samples were sequenced, considering three tissues (L, Tel and Hyp), two independent factors (stressed vs. unstressed) and (FLX treatment vs vehicle control), and six experimental replicates for each studies condition. Standard single-read mRNA libraries were built using the TruSeq Stranded mRNA Library Prep kit (Cat. # 20020595, Illumina) with 150 ng of total RNA extracts. The poly-A enrichment technique was used to select mRNA. Thereafter, all mRNAs were fragmented, converted to cDNA using random hexamers, and indexed using TruSeq RNA CD Idx (Cat. # 20019792, Illumina) for downstream multiplex analysis. The prepared libraries were assessed using Agilent 4150 TapeStation system (Cat. # G2992AA, Agilent, Santa Clara, CA, USA). The approximate band size was 260 bp for each prepared library. The indexed libraries were normalized to 10nM and pooled in equal volumes. Sequencing was done using NSQ 500/550 Hi Output KT v2.5 (Cat. # 20024906, Illumina) kits with 75 cycle cartridges. Each pooled library was run on two separate flow cells. Read alignment, as well as gene mapping to the zebrafish reference genome (GRCz11; GCA_000002035.4) was performed using STAR (42). An average of 18 million reads was mapped across different samples (range considering all samples: 12-24 million reads).

For quality assessment, QA/QC plots were generated from BAM files using Qorts (43). Furthermore, raw counts were transformed to logarithmic pseudo counts, and the boxplot functions were used to visualize between sample distribution of pseudo counts for outlier identification. Boxplots showed similar distribution of pseudo counts between samples did not reveal outlier samples

Differentially expressed genes (DEGs) analysis was performed using the DESeq2 package (44). Given our study design had FLX exposure and stress status as factors, the DESeq function was used for comparing stressed and unstressed groups considering the FLX exposure. This function also reported an adjusted p-value (padj) based on the Benjamini-Hochberg procedure, which controls the false discovery rate (FDR). The threshold to identify DEGs was an adjusted *p* value ≤ 0.05 and a fold change (FC) ≥1.2, which has been reported as the optimal FC for nervous system transcriptomic analysis (45). A hierarchal clustering (**Figure 2**) and a principle component analysis (PCA) (**Supplementary Figure 3**) was performed on the differentially expressed set of genes to further evaluate and compare the transcription patterns between treatment conditions.

### Ingenuity Pathway Analysis and Network Predictions

The lists of DEGs were analyzed using Qiagen Ingenuity Pathway Analysis (IPA; Qiagen, Hilden, Germany) to identify enrichment of canonical pathways among the differentially expressed genes. Since IPA does not have zebrafish genome annotation currently, the gene Ensembl IDs were converted to human orthologs using ID converter on DAVID (The Database for Annotation, Visualization and Integrated Discovery; https://david.ncifcrf.gov/tools.jsp). The duplicated paralogue genes in the zebrafish genome were assigned similar *Homo sapiens* orthologue Ensemble ID. All annotations reported here for pathways, networks, transcription regulators, and individual genes are according to *Homo sapiens* orthologue annotations.

A total of 27,162 (85%) gene IDs were successfully mapped by IPA. To avoid losing sensitive information, no attempt to delete or adjust duplicated probes was made. Therefore, the IPA function to assign a Z-score for activation or inhibition of any canonical pathway was not used. All analysis reported here is solely based on gene enrichment in canonical pathways. The IPA network prediction tool was used to cluster affected genes in functional molecular networks. This functionality was also used to determine potential upstream transcription regulators driving the observed alterations in the DEGs.

## Results

### Developmental Exposure to Fluoxetine Alters Gene Transcription Patterns in the Central Nervous System of Larval and Adult Zebrafish

**Figure 1:**
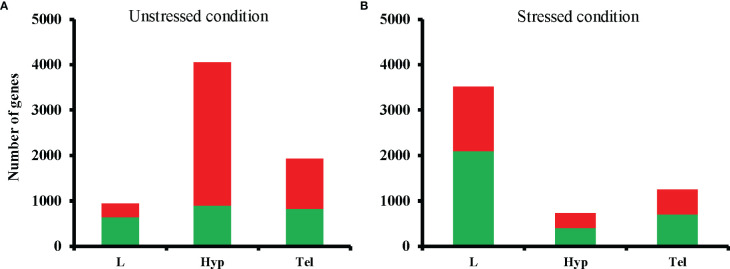
Total numbers of differentially expressed genes (DEGs) in fluoxetine-exposed groups compared to respective controls in unstressed **(A)** and stressed **(B)** conditions. The total numbers of DEGs are shown in a stacked format with downregulated and upregulated genes color-coded in green and red, respectively. L, larval head; Tel, telencephalon; Hyp, hypothalamus.

Our transcriptomic analysis showed that a total of 4456, 3172, and 5778 genes were significantly affected by developmental exposure to FLX in larvae (L), adult telencephalon (Tel), and adult hypothalamus (Hyp), respectively, regardless of stress condition. The total number of up-and down-regulated genes following developmental exposure to FLX in unstressed condition was 941, 1927, and 5055 for L, Tel, and Hyp, respectively (**Figure 1**). In the unstressed state, 68% of DEGs were downregulated and 32% were upregulated in the FLX exposed larvae (unstr-FLX-L). Interestingly, this ratio was reversed in the same unstressed condition in Hyp (unstr-FLX-Hyp), with 63% and 37% of genes upregulated and downregulated, respectively. For adult Tel in the unstressed condition (unstr-FLX-Tel), 57% and 43% of genes were upregulated and downregulated, respectively. The total number of up-and down-regulated genes following developmental exposure to FLX in the stressed condition was 3515, 1245, and 723 for L, Tel, and Hyp, respectively (**Figure 1**). In the stressed state, the ratio of up-and down-regulated genes was similar across all samples. The stressed larvae group (str-FLX-L) developmental exposure to FLX resulted in downregulation of 60% of the affected genes; the remaining 40% were upregulated. In the Hyp of stressed adult zebrafish (str-FLX-Hyp), 44% and 56% of genes were upregulated and downregulated, respectively, following developmental exposure to FLX. Moreover, 43% and 57% of genes were upregulated and downregulated in the str-FLX-Tel group, respectively (**Figure 1**). Global transcriptional responses for each condition relative to matched controls are shown **Supplementary Figure 4**.

**Figure 2:**
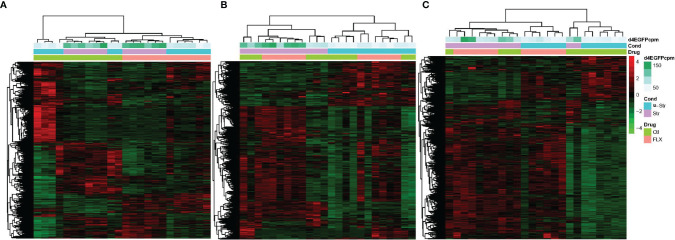
The effect of developmental fluoxetine exposure on transcriptome patterns in larval and adult male zebrafish central nervous system in the unstressed and stressed conditions. Hierarchical clustering (Spearman correlation) is shown for all 24 samples (stressed, unstressed, fluoxetine-exposed, and control ethanol-exposed) in a single pooled tissue sample (i.e., larval head, hypothalamus, and telencephalon) and their relation to FLX and stress. **(A)** FLX-exposed larvae compared to the control larvae. **(B)** telencephalon from adults exposed to FLX in the early life developmental stage compared to control adults exposed to vehicle compound (ethanol) in the early life developmental stage. **(C)** hypothalamus from adults exposed to FLX in the early life developmental stage compared to control adults exposed to vehicle compound (ethanol) in the early life developmental stage. The Y-axis consists of all genes with significant fold change (FDR ≤0.05 and FC≥ 1.2) in the treatment groups relative to the control groups. The count per million (CPM) of the reads associated with the green fluorescent protein (d4eGFP) transgene in the SR4G zebrafish line is shown. The different shades of green show different CPM. Red: up-regulation, green: down-regulation. Blue: unstressed condition, Lavender: stressed condition, Light Green: control (ethanol), Pink: Fluoxetine.

Hierarchical clustering revealed the major effects of treatment (FLX vs control) and stress status (unstressed vs stressed) on gene expression patterns (**Figure 2**). For larvae and adult Hyp, treatment (FLX vs control) was a primary driver of clustering, with subclustering based on stress status. For Tel, the stress status (unstressed vs stress) divided the main clusters (**Figure 2**). The replicates within stressed and unstressed groups were highly correlated (tightly clustered) in FLX exposed groups across all tissue samples. In contrast, three samples from the unstressed larval control groups had highly similar expression patterns that were quite distinct from the rest of the controls in this group and were on a branch of their own.

### Enrichment of Pathways Associated With Neurotransmitter Signaling and Response to Steroid Hormones Supports a Life-Long Effect of Brief Developmental Exposure to FLX

**Figure 3:**
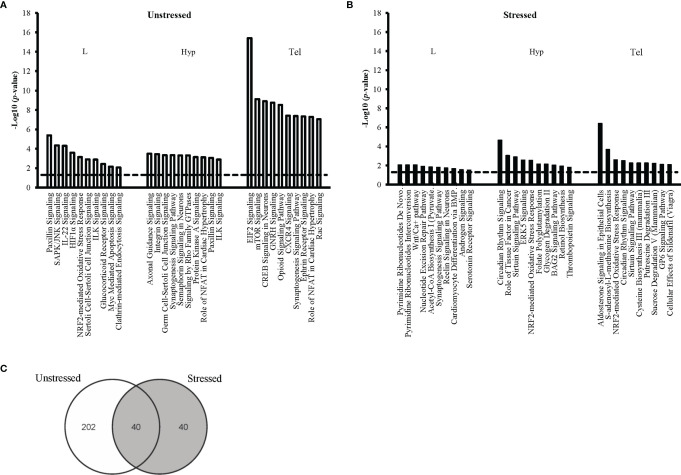
Top ten affected canonical pathways following early-life exposure to fluoxetine. The ten pathways with the highest level of significance (i.e. lowest p-value) for each target tissue are shown in larval heads (L), hypothalmus (Hyp), and telencephalon (Tel) in the unstressed **(A)** and the stressed **(B)** conditions. The y-axis presents the p-value associated with each pathway on a negative logarithmic scale (-log p-value). The horizontal dash line marks the negative logarithmic value related to the significance level p ≤0.05 [-log10(*p*)= 1.3]. Each bar shows a canonical pathway identified from the gene cluster enrichment function of IPA. The analysis was performed after converting the gene Ensembl ID of zebrafish (*Danio rerio*) to human (*Homo sapiens*) orthologues. Only genes with significant fold change (FC≥1.2; FDR ≤ 0.05) were used for pathway enrichment analysis. **(C)** The Venn diagram shows the number of all significantly affected pathways in all tissue samples from both the unstressed and the stressed conditions.

Enrichment analysis in IPA was performed to identify the pathways affected by developmental exposure. The canonical pathways were ranked based on their p-value (p ≤0.05) within each condition. We ranked the most significantly affected pathways within each tissue sample. The top 10 pathways for each of the six main groups are shown for unstressed (**Figure 3A**) and stressed conditions (**Figure 3B**). Our data showed that the cholesterol biosynthesis was significantly dysregulated in the unstressed larvae by developmental exposure to FLX; with eight of the ten mostly affected pathways were associated with cholesterol metabolism (**Figure 3A**). Also, several pathways associated with neural cell signaling, neurotransmitter signaling, and regulation of synaptic junctions populated the highly affected pathways in both Hyp and Tel of the unstressed adults (**Figure 3A**).

In the stressed groups, DNA metabolism (4 out of 10 pathways) and neurotransmitter signaling (3 out of 10 pathways) were most significantly affected by developmental exposure to FLX in the stressed larvae (**Figure 3B**). In the Hyp and Tel of stressed adults’ pathways associated with neural cell-signaling, circadian rhythm, and energy metabolism were populating the mostly affected pathways; for example, Circadian Rhythm Signaling, Sirtuin Signaling Pathway, and Aldosterone Signaling, (**Figure 3B**).

We studied the life-long effects of FLX by tracing pathways that were significantly affected in all tissue samples (L, Hyp, and Tel) or at least in one of the adult samples (Hyp or Tel). This was performed separately for unstressed (**Supplementary Tables 1**, **3**) and stressed (**Supplementary Tables 2**, **4**) conditions. For example, fifteen pathways were affected in all studied samples in the unstressed conditions: including glucocorticoid receptor signaling pathway, Aldosterone signaling in epithelial cells, IL-22 signaling, ILK, signaling, and Myc mediated apoptosis signaling. (**Supplementary Table 1**). Moreover, 47 pathways showed dysregulation in both Tel and Hyp of the unstressed adults which were exposed to FLX during their embryonic development including GNRH signaling, CREB signaling in neurons, Axonal guidance signaling, NF_κ_B signaling, STAT3 pathway, and synaptogenesis signaling pathway (**Supplementary Table 1**). Also, 14 more pathways were only affected in the Hyp tissue of the unstressed adults with another 160 canonical pathways dysregulated in the Tel tissue of the unstressed adults after developmental exposure to FLX (**Supplementary Table 3**). A similar result was observed in the stressed groups however, to a lesser extent. We observed 8 pathways dysregulated in the adult stress groups in Hyp and Tel; including Circadian Rhythm signaling, Sirturin signaling pathway, BAG2 signaling pathway, and Unfolded protein response (**Supplementary Table 2**). Also, 32 more pathways were only affected in the Hyp tissue of the stressed adults with another 26 canonical pathways dysregulated in the Tel tissue of the stressed adults after developmental exposure to FLX (**Supplementary Table 4**). Careful analysis showed that the top ten pathways within each group (**Figure 3**) were amongst pathways showing life-long dysregulation upon developmental exposure to FLX (**Supplementary Tables 1–4**).

Our findings showed that there were more significant affected pathways overall in the unstressed groups relative to the stressed groups in both FLX-exposed larvae and FLX-exposed adults (**Figure 3C** and **Supplementary Tables 1–4**). Moreover, amongst the 40 pathways that were affected in both unstressed and stressed conditions more pathways were significantly affected (based on *p*-value) in the unstressed treatment groups compared to the stressed groups (**Supplementary Table 8**).

### Upstream Transcriptional Regulators Associated With the Stress Response and Circadian Rhythm Following Developmental Exposure to FLX

An upstream regulator analysis was conducted in IPA to identify the potential key transcriptional regulators that drive the gene dysregulation observed (**Table 1**). We found enrichment of a higher number of transcriptional regulators by FLX in zebrafish larvae in unstressed condition than the other groups. It was noted that CLOCK, a key transcription factor in regulating circadian rhythm, was affected in both larvae and adults and was more affected in stressed condition (unstr-FLX-L, p=0.02; str-FLX-L, p=0.04; str-FLX-Hyp, p=5^E-06^; str-FLX-Tel, p=9^E-06^). Such significant effects were not observed in the Hyp and the Tel in the unstressed condition (unstr-FLX-Hyp, p>0.05; unstr-FLX-Tel, p>0.05). Other transcriptional regulators that were observed in the adult Hyp and Tel in stressed fish include: NR3C1 (str-FLX-L, p=0.03; str-FLX-Tel, p=0.02), MAT1A (str-FLX-L, p=0.002; str-FLX-Tel, p=0.02), NPC1 (str-FLX-Hyp, p=0.02; str-FLX-Tel, p=0.04), SIRT1 (str-FLX-Hyp, p=0.02; str-FLX-Tel, p=0.02), and NCOA1 (str-FLX-Hyp, p=6^E-04^; str-FLX-Tel, p=0.0001). Moreover, in the unstressed condition we observed several transcriptions regulators were affected in adult Hyp and Tel; for example, CPT1C (unstr-FLX-Hyp, p=0.03; unstr-FLX-Tel, p=0.002), SREBF1 (unstr-FLX-L, p=0.02; unstr-FLX-Tel, p=8^E-04^), and SREBF2 (unstr-FLX-L, p=0.001; unstr-FLX-Tel, p=2^E-04^), SCAP (unstr-FLX-L, p=0.01; unstr-FLX-Tel, p=0.002), and PPARGC1A (unstr-FLX-L, p=0.009; unstr-FLX-Tel, p=0.004).

**Table 1:** Upstream regulators associated with the genes altered by developmental exposure to fluoxetine in zebrafish.

Upstream regulator	Gene ontology: Biological process	Unstressed					
		L	Hyp	Tel	L	Hyp	Tel
CLOCK	circadian regulation of gene expression						
NPC1	cholesterol metabolic process						
SIRT1	circadian regulation of gene expression						
NFE2L2	protein catabolic process						
RICTOR	TOR signaling						
CPT1C	fatty acid beta-oxidation						
CCL4	immune response						
PPARD	cholesterol metabolic process						
DERL1	positive regulation of protein binding						
NR0B2	cholesterol metabolic process						
PML	circadian regulation of gene expression						
GCK	cellular glucose homeostasis						
PPARGC1A	circadian regulation of gene expression						
SCAP	cholesterol metabolic process						
SREBF1	cholesterol metabolic process						
SREBF2	cholesterol metabolic process						
ABCA1	cholesterol metabolic process						
SIRT3	histone H3 deacetylation						
NCOA1	histone H4 acetylation						
STAT3	negative regulation of neuron death						
NR3C1	stress response						
MAT1A	methylation						

Only significant molecules (p ≤ 0.05) are shown in the blue gradient. Gray represents no significant different from controls. L, larval heads; Hyp, hypothalamus; Tel, telencephalon. Gene ontology is associated with Homo sapiens orthologs.

### Developmental Exposure to FLX Dysregulates Genes Associated With Lipid Metabolism, Nervous System Development and Behavior

The top 5 networks in each tissue sample and their IPA functional categories are shown (**Table 2**). The scoring is based on the number of genes involved and the internal scoring algorithm of IPA. Our findings suggest that several predicted networks were affected in adults upon developmental exposure to FLX. For example, genes involved in “lipid metabolism” and “molecular transport” were significantly enriched in larvae and adult in both stressed and unstressed condition. Moreover, networks involved in “nervous system development” and “endocrine system development” were affected in the adult Hyp and Tel.

**Table 2:** Top 5 predicted networks affected by developmental exposure to fluoxetine.

Group	Score	# Genes	Functional clustering*
**L (unstressed)**	43	35	Lipid Metabolism, Molecular Transport
	14	19	Hepatic System Disease, Liver Steatosis
	13	18	Lipid Metabolism, Molecular Transport
	11	17	Lipid Metabolism, Molecular Transport
	10	16	Lipid Metabolism, Molecular Transport
**Hyp (unstressed)**	24	35	Drug Metabolism, Small Molecule Biochemistry
	24	35	Lipid Metabolism, Molecular Transport
	24	35	Endocrine System Development and Function
	24	35	Hepatic System Disease, Liver Steatosis
	11	26	Cancer, Gene Expression, Organismal Injury and Abnormalities
**Tel (unstressed)**	34	35	Lipid Metabolism, Small Molecule Biochemistry
	34	35	Cellular Development, Cellular Growth and Proliferation
	13	16	Cell Morphology, Cellular Function and Maintenance
	11	16	Cell-To-Cell Signaling and Interaction, Cellular Function and Maintenance
	10	19	Cellular Compromise, Organismal Injury and Abnormalities
**L (stressed)**	29	35	Lipid Metabolism, Molecular Transport
	29	35	Lipid Metabolism, Small Molecule Biochemistry
	12	16	Developmental Disorder, Hereditary Disorder, Metabolic Disease
	9	21	Lipid Metabolism, Molecular Transport
	9	17	Behavior, Nervous System Development and Function
**Hyp (stressed)**	12	16	Lipid Metabolism, Molecular Transport
	12	16	Lipid Metabolism, Molecular Transport
	12	16	Lipid Metabolism, Molecular Transport
	12	16	Behavior, Nervous System Development and Function
	9	14	Lipid Metabolism, Molecular Transport
**Tel (stressed)**	11	16	Lipid Metabolism, Behavior, Nervous System Development and Function
	11	17	Lipid Metabolism, Small Molecule Biochemistry
	11	17	Lipid Metabolism, Molecular Transport
	11	17	Cellular Development, Tissue Development
	11	17	Metabolic Disease, Organismal Injury and Abnormalities

*Gene ontology is associated with Homo sapiens orthologs. L, larval heads; Hyp, hypothalamus; Tel, telencephalon.

### FLX Dysregulates Gene Expression in Both Larvae and Adult Zebrafish

**Figure 4:**
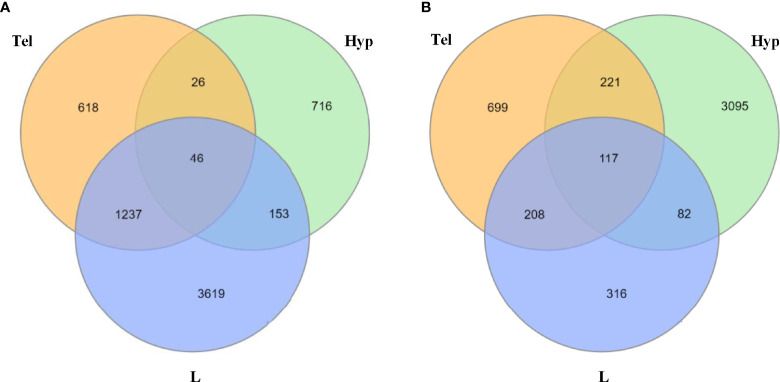
Number of common dysregulated genes in larvae and adult zebrafish upon developmental exposure to fluoxetine in stressed and unstressed conditions. **(A)** Venn diagram illustrating the common differentially expressed genes between larval heads and adult telencephalon and hypothalamus in the unstressed condition. **(B)** Venn diagram illustrating the common differentially expressed genes between larval heads and adult telencephalon and hypothalamus in the stressed condition. All of the significantly dysregulated genes (up or downregulated) were included in this comparison (FC≥1.2; p ≤ 0.05). L, larvae; Hyp, hypothalamus; Tel, telencephalon.

Many genes were dysregulated in both larvae and adults. We report that 46 and 117 genes were dysregulated (up- or down-regulated) upon developmental exposure to FLX in all tissue samples (L, Hyp, Tel) in the unstressed and stressed conditions, respectively (**Figure 4**). The associated functional enrichment of these genes based on Gene Ontology annotations of *Homo sapiens* orthologues are shown in **Supplementary Tables 5**,**6** for the unstressed and stress conditions, respectively. Downregulation predominated in the unstressed larvae (unstr-FLX-L, 38 out of 46 genes) and upregulation predominated in the unstressed adults (unstr-FLX-Hyp, 34 out of 46; unstr-FLX-Tel, 34 out of 46). In contrast, upregulation was more prevalent in the stressed larvae (str-FLX-L, 95 out of 117 genes) and downregulation was the main feature in the stressed adults (str-FLX-Hyp, 91 out of 117; str-FLX-Tel, 91out of 117). Interestingly, for most of the genes in the unstressed and stressed responses, the direction of the gene expression change in larvae was opposite to that in adults; 93% of genes and 82% of genes for unstressed and stressed conditions, respectively.

### Life-Long Effects of Developmental Exposure to FLX on Genes Associated With Depression, Neuroplasticity and Epigenetics

To evaluate the potential life-long impact of fluoxetine exposure on several pathways we clustered the pathways which were affected in both larval stage and adult stage (from either Hyp or Tel or both). Any given pathway that was significantly dysregulated compared to the control samples, given the description above, in either stressed or unstressed conditions was considered to be a potential within-generation affected pathway. A list of 512 genes associated with the major depressive disorder was extracted from the DisGeNET database (46). Our transcriptome analysis shows that more than 80% of the orthologues of these genes were affected by developmental exposure to FLX at least in one of the studied tissue samples (**Supplementary Table 6**). The main dysregulation was in adult Hyp in the unstressed condition, with 271 depression-associated genes significantly affected. Among these, 73% were upregulated upon developmental exposure to FLX. Examples of genes downregulated in adult brain following developmental exposure to FLX in the unstressed condition include: *nrgn* (unstr-FLX-Hyp: FC= -1.4, p=0.04; unstr-FLX-Tel: FC= -1.3, p=0.008), *fabp2* (unstr-FLX-Hyp: FC=-1.6, p=0.02; unstr-FLX-Tel: FC=-2, p=0.04), *klf11a* (unstr-FLX-Hyp: FC=-1.4, p=0.007; unstr-FLX-Tel: FC= -1.45, p= 0.005), and *pvalb6* (unstr-FLX-Hyp: FC=-1.2, p=0.01; unstr-FLX-Tel: FC= -1.6, p= 0.03). Similar downregulation was also observed in the stressed adult zebrafish; for example, *abcc1* (str-FLX-Hyp: FC= -1.7, p= 1.5^E0-8^; str-FLX-Tel: FC= -1.5, p= 1.4^E0-12^), *fkbp4* (str-FLX-Hyp: FC= -1.4, p=1.2^E0-7^; str-FLX-Tel: FC=-1.3, p=9^E-016^), *pclob* (str-FLX-Hyp: FC= -1.4, p= 0.04; str-FLX-Tel: FC=-1.2, p= 0.02), *srrt* (str-FLX-Hyp: FC= -1.4, p= 0.00002; str-FLX-Tel: FC= -1.3, p= 0.002), and *tspo* (str-FLX-Hyp: FC= -2, p= 7^E0-7^; str-FLX-Tel: FC=-1.7, p= 2^E0-9^).

We also found that several genes associated with development of depressive disorders were upregulated in adult brain following developmental exposure to FLX. For example, *elovl5* (unstr-FLX-Hyp: FC= 1.7, p=0.001; unstr-FLX-Tel: FC= 1.9, p= 0.04; str-FLX-Hyp: FC=1.9, p= 9^E0-6^; str-FLX-Tel: FC=1.9, p=0.008)*, tefb* (unstr-FLX-Hyp: FC=2, p= 4^E0-6^; unstr-FLX-Tel: FC= 1.5, p= 0.002; str-FLX-Hyp: FC= 1.6, p= 0.002; str-FLX-Tel: FC= 1.8, p= 7^E0-10^) and *klf15* (unstr-FLX-Hyp: FC= 2.5, p= 2^E0-7^; unstr-FLX-Tel: FC= 1.7, p= 0.002; str-FLX-Hyp: FC= 1.6, p= 0.006; str-FLX-Tel: FC= 1.4, p=0.03) were upregulated in both adult Tel and Hyp in both unstressed and stressed conditions. Several genes associated with development of depressive disorders were upregulated in adult brain following developmental exposure to FLX only in the unstressed condition; e.g., *nr3c2* (unstr-FLX-Hyp: FC= 3.12, p= 2^E0-5^; unstr-FLX-Tel: FC= 1.6, p= 0.01), *clocka* (unstr-FLX-Hyp: FC= 2, p= 2^E0-6^; unstr-FLX-Tel: FC= 1.4, p= 0.03)*, grm3* (unstr-FLX-Hyp: FC= 2.3, p= 5^E0-6^; unstr-FLX-Tel: FC= 1.8, p= 0.02), and *ntrk2a* (unstr-FLX-Hyp: FC= 1.7, p= 3^E0-12^; unstr-FLX-Tel: FC= 1.4, p= 0.006). Additionally, genes associated with development of depressive disorders were upregulated in adult brain following developmental exposure to FLX only in stressed condition, e.g., *grm2a* (str-FLX-Hyp: FC=1.2, p= 0.01; str-FLX-Tel: FC= 2, p= 1.2^E0-7^), *per1b* (str-FLX-Hyp: FC= 1.8, p= 5^E0-4^; str-FLX-Tel: FC= 1.7, p= 2^E0-8^) and *per1a* (str-FLX-Hyp: FC= 3, p= 5^E0-6^; str-FLX-Tel: FC= 2.6, p= 2^E0-7^) (**Supplementary Table 7**).

Finally, a list of genes with implication is epigenetics was extracted from the Gene Ontology database using *Homo sapiens* GO: biological process annotations. **Table 3** shows the genes that were significantly dysregulated following developmental exposure to FLX in larvae and adult zebrafish in both unstressed and stressed conditions. Upregulation of these genes in unstressed condition in =Hyp and Tel of adult zebrafish was the main effect.

**Table 3:** Life-long dysregulation of genes involved in epigenetics following developmental exposure to fluoxetine.

Gene symbole		Unstressed			Stressed			Gene ontology: Biological process*
*Homo sapiens*	*Danio rerio*	L	Hyp	Tel	L	Hyp	Tel	
*HDAC4*	*hdac4*							Chromatin remodeling
*HDAC5*	*hdac5*						
*KAT2A*	*kat2a*						
*KAT2B*	*kat2b*						
*ACTR1A*	*actr1*							G2/M transition of mitotic cell cycle
*ADARB1*	*adarb1b*							mRNA processing
*ADARB2*	*adarb2*						
*ATF2*	*atf2*							Negative regulation of transcription from RNA polymerase II promoter
*DNMT3A*	*dnmt3aa*						
*FOSB*	*fosb*						
*HDAC8*	*hdac8*						
*MECP2*	*mecp2*						
*FP565260.4*	*dnmt3bb.3*							Novel protein
*HCN1*	*hcn1*							Regulation of postsynaptic membrane potential

*Gene ontology is associated with Homo sapiens orthologs. L, larval heads; Hyp, hypothalamus; Tel, telencephalon. Green, downregulated; Red, upregulated.

## Discussion

In the current study we focused on the potential life-long impacts of early developmental exposure to FLX on the stress axis in zebrafish. We report that developmental exposure to FLX alters the transcription profile in the central nervous system of exposed larvae in both unstressed and stressed conditions. We show that this brief exposure to FLX during development resulted in distinct transcriptomic profiles in the hypothalamus and telencephalon of the adult male zebrafish compared to the control groups. Such life-long effects were seen in both the unstressed and stressed conditions. Pathway analysis revealed significant dysregulation of more than 280 pathways; many associated with circadian rhythms, hormone synthesis, neurotransmitter signaling and synaptogenesis. Moreover, the network prediction analyses performed suggested that expression of many genes involved in neural development, cell signaling, and lipid metabolism were affected. Although there are reports about the effect of FLX exposure on the transcriptome in adult whole brain (47) and larval zebrafish (larval brain, larval whole body, and embryos) (48–50), to our knowledge this is the first study to report on life-long transcriptomic changes in the central nervous system of adult male zebrafish. Previously in our lab, we reported that a similar developmental exposure to FLX caused blunted stress responses in adult male zebrafish, for at least three generations. Our findings, further support the effect of developmental exposure to FLX on the dysregulation of the stress response; evident by altered transcript levels of several genes in the stress-related pathways including glucocorticoid receptor signaling, circadian rhythm signaling, and neurotransmitter synthesis. Furthermore, with many genes and pathways associated with neurogenesis and synaptogenesis, FLX-exposure during development could potentially alter the nervous system dynamics. This could affect the adaptability or survival of fish residing in rivers receiving municipal wastewaters containing significant levels of SSRIs. Such concerns have already been raised and reviewed extensively. Moreover, the 54 µg/L dose of FLX exposure studied here is within the range detected in human fetal umbilical blood sample. Several studies both in humans and rodents indicate adverse effects of maternal exposure to SSRIs, including FLX, on behavior and anxiety-symptoms in offspring. Our findings help elucidate the potential pathways and mechanisms of such adverse effects.

Our results suggest dysregulation of transcription factors involved in circadian rhythm, stress response, cholesterol metabolism, and histone modifications. Gene expression profiles were consistent with dysregulation of the signaling pathway associated with *clocka* (orthologue to CLOCK in *Homo sapiens*) in unstressed larvae, stressed larvae, and stressed adults developmentally exposed to FLX.Circadian locomotor output cycles kaput protein (CLOCK) plays a central role in regulating circadian rhythms in all vertebrates (51). In mammals, binding of CLOCK and its paralog NPAS2, to the regulatory elements upstream of several rhythmic genes, including PER1, PER2, PER3, CRY1, and CRY2 activates their transcription (51). Several mental and metabolic disorders in humans are associated with dysregulation of CLOCK and its related genes, including major depressive disorder, bipolar disorder, schizophrenia, diabetes mellitus and obesity (52). Our results build on previous findings of disruption of CLOCK-related genes by FLX in both rodent and teleost models. Kiryanova et al. showed that developmental exposure to FLX in mice (administering 25 mg/kg/day FLX to the drinking water of the pregnant dam) disrupts the normal circadian rhythm in adolescents two months old male offspring (53). This was evident by a significantly higher number of days needed for the affected individuals to adapt to a new day-light cycle (53). Moreover, it is known that FLX exposure in zebrafish decreases the amount of melatonin production, potentially leading to altered circadian rhythm (17). Wu et al. have shown that several genes associated with circadian rhythm regulation, including *nr1d1* and *per2*, are affected by exposure to FLX (10 µg/L), amitriptyline (0.1 µg/L), and mianserin (10 µg/L) (50). In our previous study using a similar FLX exposure paradigm for zebrafish larvae, we uncovered downregulation of several genes whose mammalian orthologues are associated with stress and circadian rhythms including *fkbp5, npas4a, nr4a1, per2*, and *rorcb* (40). Similar findings are presented here in our RNAseq results. We also predicted life-long dysregulation in glucocorticoid receptor signaling (*nr3c1*) in stressed individuals. Blunted stress response following exposure to FLX has previously been reported in rodents and telosts. For example, Pawloski et al. showed that 28-days of maternal prenatal exposure to FLX in rats results in blunted corticosterone levels and reduces GR expression in the hippocampal region more prominently in adolescent male offspring (29). Giacomini et al. reported that adult male zebrafish exposed to FLX (50µg/L) for 15 days have reduced cortisol levels and a blunted stress response upon exposure to the novel tank test (54). Adult zebrafish exposed to FLX (50µg/L) have a blunted cortisol response following both physical (chasing) and chemical (osmotic shock) stress (39). A similar blunted cortisol level was reported following 15 minutes exposure to a lower concentration of FLX (1µg/L) before an osmotic stressor in adult zebrafish (55). Recently, we reported transgenerational hypocortisolism following developmental six-day exposures to FLX (54µg/L) in descendant generations for at least three generations in male zebrafish (14). We now report that such life-long or even transgenerational effects might be driven by disruption of the stress axis and other endocrine pathways in the adult Hyp Other than circadian rhythm pathways and glucocorticoid receptor signaling, we predicted dysregulation within GNRH signaling, androgen signaling, relaxin signaling, and prolactin signaling in both stressed and unstressed conditions. Dysregulation of these pathways is associated with development of depressive disorders in humans. It should be emphasized that we used IPA to determine the potential upstream transcriptional regulators (i.e., transcription factors) which may help to explain the observed transcriptomic patterns. This is a predictive algorithm that examines how many known targets of each transcription regulator are present in a given dataset, and also compares their direction of change (i.e., expression in the experimental sample(s) relative to control) to what is expected from the literature in order to predict likely relevant transcriptional regulators (56). The identification of a particular transcription factor (for example CLOCK) does not necessarily mean an altered mRNA level (i.e., transcription) of that transcription factor on the studied sample; rather it implies a global transcriptional changes in the pool of assessed genes and the involvement of a particular transcription factor. The IPA upstream regulator prediction algorithm is a powerful tool to help interpret the snapshot of the ongoing transcriptional cascade.

To date, 512 genes have been clinically associated with the development of major depressive disorder and similar ailments (46). We found that the orthologues of many of these genes were upregulated in the adult Tel and Hyp upon developmental exposure to FLX. For example, we observed dysregulation of *bdnf*, *trkb* (bdnf receptor), and *npas4* (a *bdnf* transcriptional regulator) in FLX-exposed larvae and adults. BDNF and TrKB-mediated neurogenesis and synaptogenesis are suggested as therapeutic means for FLX in humans (57). It is known that NPAS4 is significantly downregulated in the hippocampus and prefrontal cortex of SERT-/- rats, a mammalian model for mood disorders (58). Our findings suggest that FLX may have long-lasting effect on these genes well after withdrawal from drug exposure. Many studies demonstrate altered behavior or endocrine function after FLX exposure. For example, in rats, early life exposure to SSRIs reduces social behaviors and social preference later in life during the juvenile period and adulthood in both sexes (59, 60). We thus predicted that we would see effects of FLX on gene networks linked to behavior. Given the list of genes we uncovered, altered neurogenesis and neural plasticity are some of the possible processes underlying this.

The exact mechanism by which developmental exposure to FLX could alter brain programing is yet to be determined. It is known that serotonin modulates neurogenesis in different regions of the developing zebrafish brain (61). Given the well-described roles of serotonin in early brain development (5–7) and our observation of altered transcription of multiple genes and pathways associated with neurogenesis, it is possible that altered serotoninergic tone resulting from FLX exposure could modulate neurogenesis in the developing brain. One of the other hypotheses to explain the persistent effects of developmental exposure to pharmaceuticals is alterations in epigenetic markings such as DNA methylation and histone modifications. In keeping with this hypothesis, we found that FLX exposure altered the expression of a variety of genes associated with epigenetic machinery. Several genes including *dnmt3a, adarb1, adarb2, hdac4, hdac5, hdac8*, and *atf2* were significantly affected. Interestingly, upregulation of these genes in the adult brain was a predominant observation. An increasing body of evidence attributes the beneficiary response to antidepressant therapies to epigenetics (62, 63). Vatencourt et al. showed that FLX therapy transiently increases *Bdnf* expression in adult rat visual cortex (64). This is accompanied by increased H3K9 acetylation of the *Bdnf* promoter, a chromatin remodeling associated with the epigenetic marking of DNA (64). Moreover, Wang et al. showed that FLX treatment after traumatic brain injury increases the rate of hippocampal neurogenesis in mice (65). This increase in neurogenesis coincides with an increase in H3K9 acetylation and MBD-1 (methyl-CpG-binding protein 1) immunoreactive cells in the hippocampal region of treated mice (65). Robinson et al. also reported that FLX reduces the acetylation of histone 3 (H3) at the promoter region of CaMKIIa in a mouse model for anxiety-like behavior (66). This results in downregulation in CaMKIIa, which improves the mood and behavior of mice (66). Zaidan et al. demonstrated that pre-gestational FLX treatment of the dam enhances ADAR enzyme expression, a family of RNA editing enzymes associated with epigenetic marking, in the offspring amygdala in rats (67). This change was associated with the reversal of the effects of pre-reproductive stress of dam on adult offspring’s social behavior (67). The alteration in the transcription profile of epigenetic-machinery genes might be partially responsible for the observed life-long effect of FLX. Further studies including histological assessments of brain sections, methylation profiling of promoter regions in genes of interest, or other epigenetic markings (histone acetylation, microRNA s, etc) are required to determine the exact nature of this relationship, and the mechanisms underlying the long-term effects of developmental FLX exposure.

In our study, we showed that the *egfp* transgene mRNA levels could be used to mark the desired samples and track changes compared to other genes in a sequencing data set. We have recently showed that there is a significant positive correlation between whole-body cortisol levels and whole-body eGFP *egfp* in 7 dpf SR4G zebrafish larvae (40). Such a positive relationship is also evident in our sequencing data for zebrafish larvae where all stressed samples clustered together in both FLX-exposed and vehicle-exposed groups. Although it is reported that the eGFP expression is ubiquitous in the SR4G line (38), the level of *egfp* mRNA is highly dependent on the amount of glucocorticoid receptor (GR) expression in any given tissue. As the expression of GR is not similar in all cells and tissues, therefore, one should expect difference in eGFP expression in different tissues or cell populations in the SR4G line. In our study, we noted a somewhat higher levels of variation in *egfp* in the unstressed-vehicle-exposed samples compared to stressed samples and FLX-exposed samples. Several factors could contribute the observed variations. It is known that the baseline cortisol level is differ between free-living individuals as the cope with daily challenges. Viewed under the lens of Romero’s Reactive Scope Model (68) baseline variations in the control groups are to be expected, and are indeed normal. One action of FLX was to reduce variations between the groups through clear suppression of cortisol and genes regulated by the glucocorticoid. Moreover, we observed clustering (i.e., two subgroups) in the unstressed larval samples. Some of the pathways (data not shown) that appeared to differ in the subgroups were related to muscle contraction, collagen formation, keratinization, among other structural aspects. This suggests some variation in the overall developmental stage in head tissues at day 7 when we are examining the snapshot transcriptomic view of these rapidly developing larvae. The pooled tissues 22-28 larval heads are a mix of samples from undifferentiated males and females, as sexing is not feasible for 7-dpf larvae. On the other hand, the study on adult brain was performed on only male individuals. It will be important in new experiments to investigate the variation amongst control larvae at these early developmental stages. 

In conclusion, we provide evidence that developmental exposure to FLX leads to life-long changes in the expression of hundreds of genes associated with critical regulatory networks, canonical pathways and development/neurological diseases. We also report many similar findings to mammalian models, which further supports the benefits of using zebrafish in neuropsychological studies of anti-depressants’ effects. Overall, we found the developmental FLX exposure resulted in the disruption of several pathways associated with neuroendocrine signaling, stress response and circadian rhythm, all of which are implicated in depressive disorders in humans. The observed alterations of epigenetic modifying enzymes and genes involved in the neurogenesis that persisted to adulthood could explain the dysregulation observed in these major hypothalamic pathways. Our study provides insight into the potential pathways responsible for the long-lasting effects of FLX.

## Data Availability Statement

The original contributions presented in the study are publicly available. This data can be found here: NCBI GEO repository, GSE141144.

## Ethics Statement

The animal study was reviewed and approved by University of Ottawa Animal Care and Veterinary Services Ethic committee.

## Author Contributions

AN designed the study and methodology, conducted most of the experiments and analyses and prepared the manuscript. RG performed the RNA sequencing and associated analyses. CL performed MS analysis. CY assisted with the RNA sequencing experimental design and edited the manuscript. VT supervised and funded the research, helped with the design of the study and manuscript writing. All authors contributed to the article and approved the submitted version.

## Funding

Supported by the NSERC Discovery Program (VT), University of Ottawa Research Chair in Neuroendocrinology (VT) and Health Canada (CY).

## Conflict of Interest

The authors declare that the research was conducted in the absence of any commercial or financial relationships that could be construed as a potential conflict of interest.

## Publisher’s Note

All claims expressed in this article are solely those of the authors and do not necessarily represent those of their affiliated organizations, or those of the publisher, the editors and the reviewers. Any product that may be evaluated in this article, or claim that may be made by its manufacturer, is not guaranteed or endorsed by the publisher.

## Supplementary Material

Supplementary Figure 1 Experimental design used to study the life-long effects of developmental exposure to fluoxetine in zebrafish. Zebrafish larvae (each petri dish containing 22-28 larvae) daily exposed daily to fluoxetine (54µg/L) or control (ethanol 0.005%) from 0 to 6 dpf. Each group was further divided into stressed (Str) and unstressed (Unstr) based on the handling stress routine described elsewhere in this manuscript. A pooled sample of larval heads (22-28 larvae, 7 dpf) were prepared. Each experiment was conducted in 6 experimental replicates. Several subgroups of developmentally exposed and not-exposed larvae were raised to adulthood (6 months of age) in separate tanks. They were further divided into stressed and unstressed based on the handling stress routine described elsewhere in this manuscript. The telencephalon and hypothalamus were harvested from 5 male individuals in each group and pooled in a single sample tube. A total of 6 experimental replicates was assessed. The statistical comparison for the RNAseq study was performed between unstressed and stressed condition considering the fluoxetine exposure background.

Supplementary Figure 2 The net handling stress protocol for larvae and adult zebrafish. The top panel depicts the handling stress for 7 dpf larvae and consisted of two 1-minute net stress and room air exposure separated by a 3-minutes resting and free-swimming episode. Larvae were sacrificed 60 minutes after the last net stress, and larval heads were collected and 22-28 pooled in a single 2 ml sample tube. The bottom panel depicted the handling stress protocol for adult zebrafish and consisted of two 3-minute net stressing and room air exposure separated by a 3-minutes resting and free-swimming. Adult zebrafish were sacrificed 60 minutes after the last net stress, and telencephalon and hypothalamus were collected. The red dashed line depicts the incision sites on the brain anatomy to acquire telencephalon and hypothalamus samples. The telencephalon and hypothalamus from five adult fish were pooled in a single 2 ml sample tube. All experiments were performed with six experimental replicates (N=6).

Supplementary Figure 3 Principle component analysis of the mapped transcriptome in fluoxetine-exposed groups compared to the controls in unstressed and stressed conditions. The PCA 1 (x-axis) and PCA 2 (y-axis) in the three studied tissues (L, Hyp, and Tel) in the unstressed and stressed conditions are shown. Only genes with FC≠≥ 1.2 are depicted here. The blue circles represent the FLX exposed groups, and the red circles represent the control groups. The size of the circles defines the stress status.

Supplementary Figure 4 Volcano plots of the mapped transcriptome in fluoxetine-exposed groups compared to the controls in unstressed and stressed conditions. The volcano plots in the three studied tissues in the unstressed and stressed conditions are shown. The x-axis shows the fold change (FC) on a logarithmic scale. Only genes with FC≥ 1.2 are depicted here. The y-axis shows the level of significance on a negative logarithmic scale. The scales of the y-axis are different for unstressed and stressed conditions to illustrate the data points clearly. The blue line shows the adjusted *p*-value ≤ 0.05 (equal to 1.3 on a negative logarithmic scale). The genes with significant FC are demonstrated as red dots above the blue threshold line. The genes with the most significant fold change and the highest significance are labeled in each graph. L, larval heads; Hyp, hypothalamus; Tel, telencephalon.
