# Supplementary figures and images for: Brief Developmental Exposure to Fluoxetine Causes Life-Long Alteration of the Brain Transcriptome in Zebrafish
Source: Front Endocrinol (Lausanne). 2022 Apr 28;13:847322. doi: 10.3389/fendo.2022.847322 (PMC9097470; doi:10.3389/fendo.2022.847322)

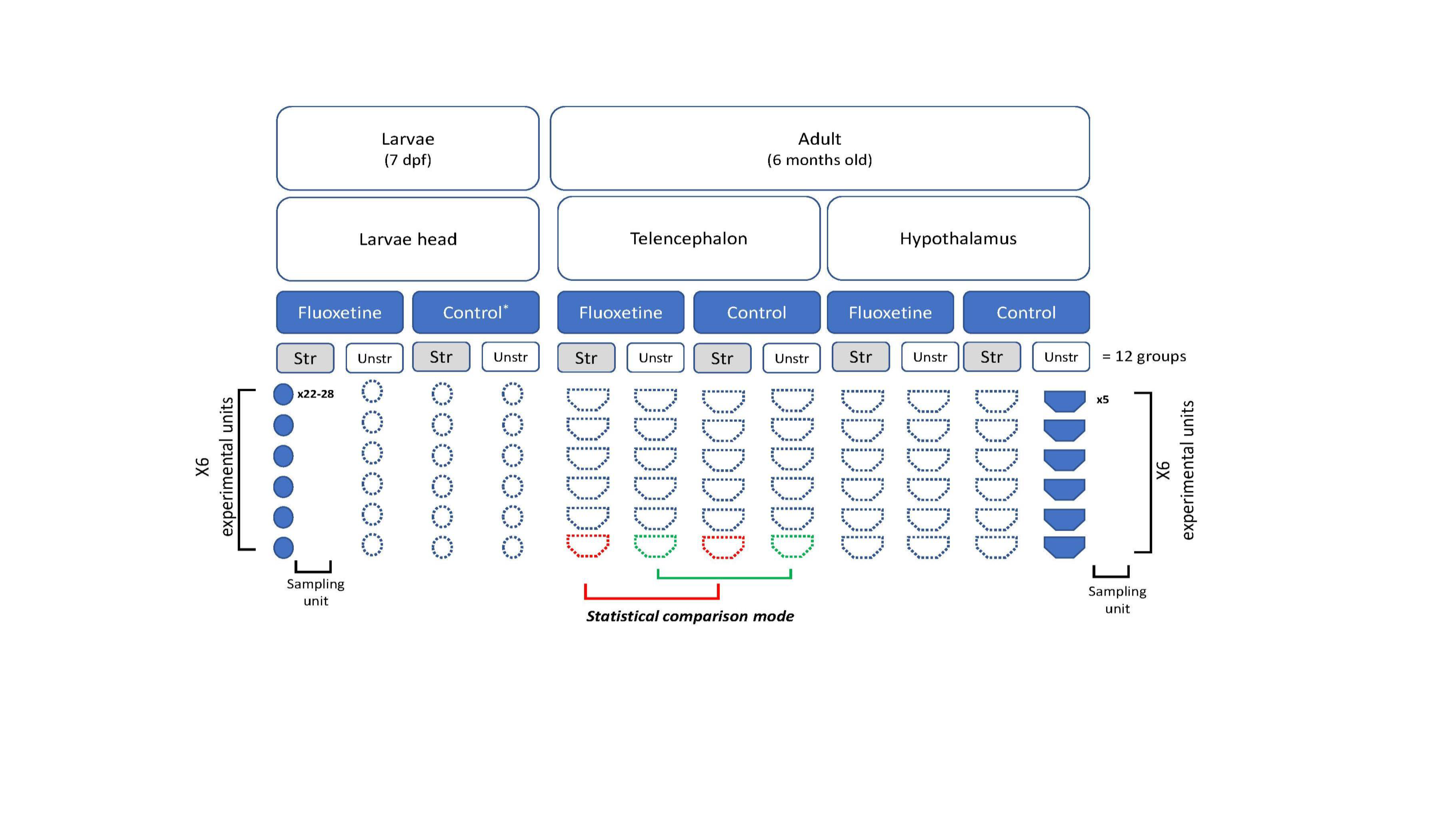

Supplement: Supplementary Figure 1 — Experimental design used to study the life-long effects of developmental exposure to fluoxetine in zebrafish. Zebrafish larvae (each petri dish containing 22-28 larvae) daily exposed daily to fluoxetine (54µg/L) or control (ethanol 0.005%) from 0 to 6 dpf. Each group was further divided into stressed (Str) and unstressed (Unstr) based on the handling stress routine described elsewhere in this manuscript. A pooled sample of larval heads (22-28 larvae, 7 dpf) were prepared. Each experiment was conducted in 6 experimental replicates. Several subgroups of developmentally exposed and not-exposed larvae were raised to adulthood (6 months of age) in separate tanks. They were further divided into stressed and unstressed based on the handling stress routine described elsewhere in this manuscript. The telencephalon and hypothalamus were harvested from 5 male individuals in each group and pooled in a single sample tube. A total of 6 experimental replicates was assessed. The statistical comparison for the RNAseq study was performed between unstressed and stressed condition considering the fluoxetine exposure background. [file Image_1.tiff]

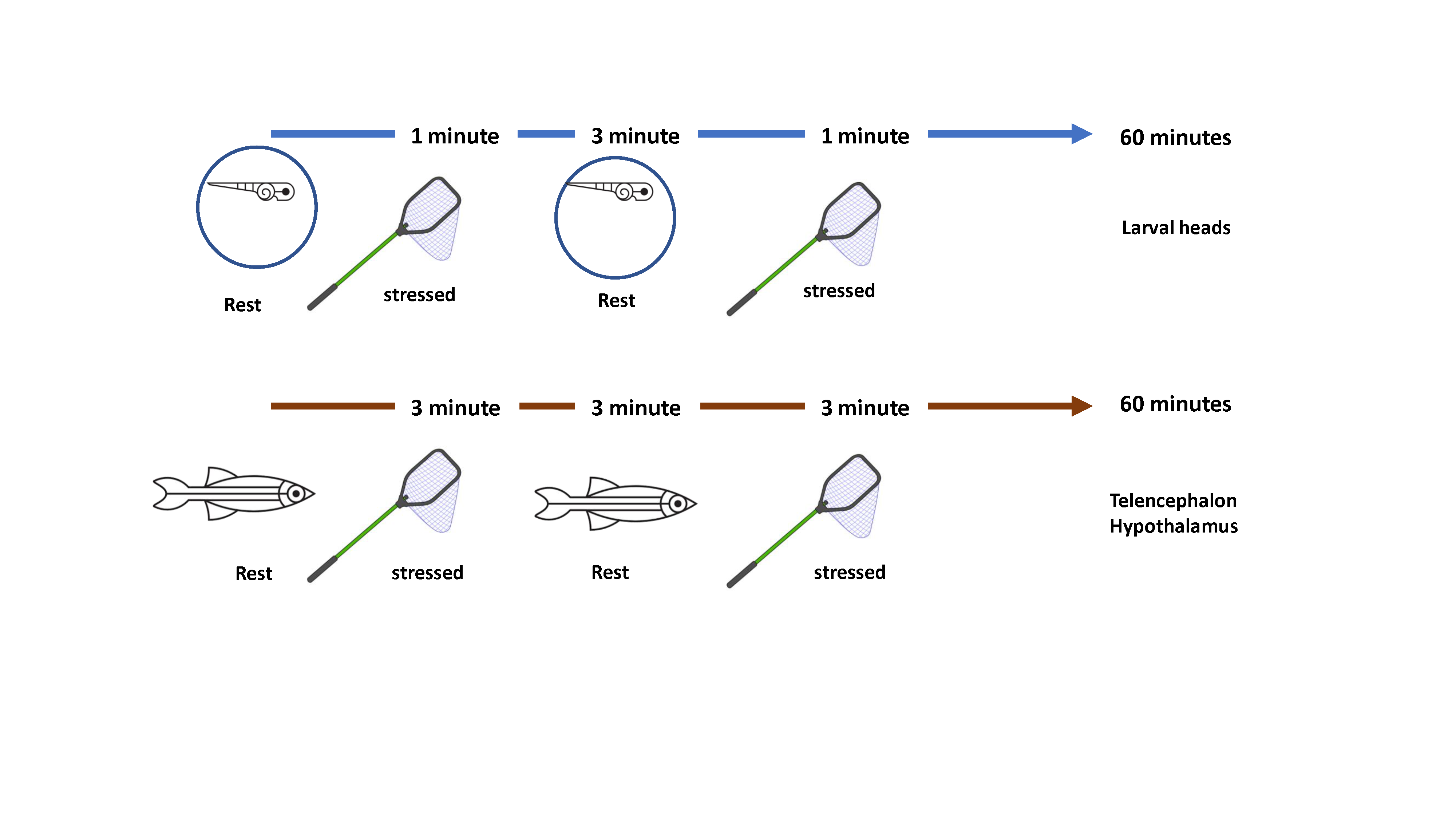

Supplement: Supplementary Figure 2 — The net handling stress protocol for larvae and adult zebrafish. The top panel depicts the handling stress for 7 dpf larvae and consisted of two 1-minute net stress and room air exposure separated by a 3-minutes resting and free-swimming episode. Larvae were sacrificed 60 minutes after the last net stress, and larval heads were collected and 22-28 pooled in a single 2 ml sample tube. The bottom panel depicted the handling stress protocol for adult zebrafish and consisted of two 3-minute net stressing and room air exposure separated by a 3-minutes resting and free-swimming. Adult zebrafish were sacrificed 60 minutes after the last net stress, and telencephalon and hypothalamus were collected. The red dashed line depicts the incision sites on the brain anatomy to acquire telencephalon and hypothalamus samples. The telencephalon and hypothalamus from five adult fish were pooled in a single 2 ml sample tube. All experiments were performed with six experimental replicates (N=6). [file Image_2.tif]

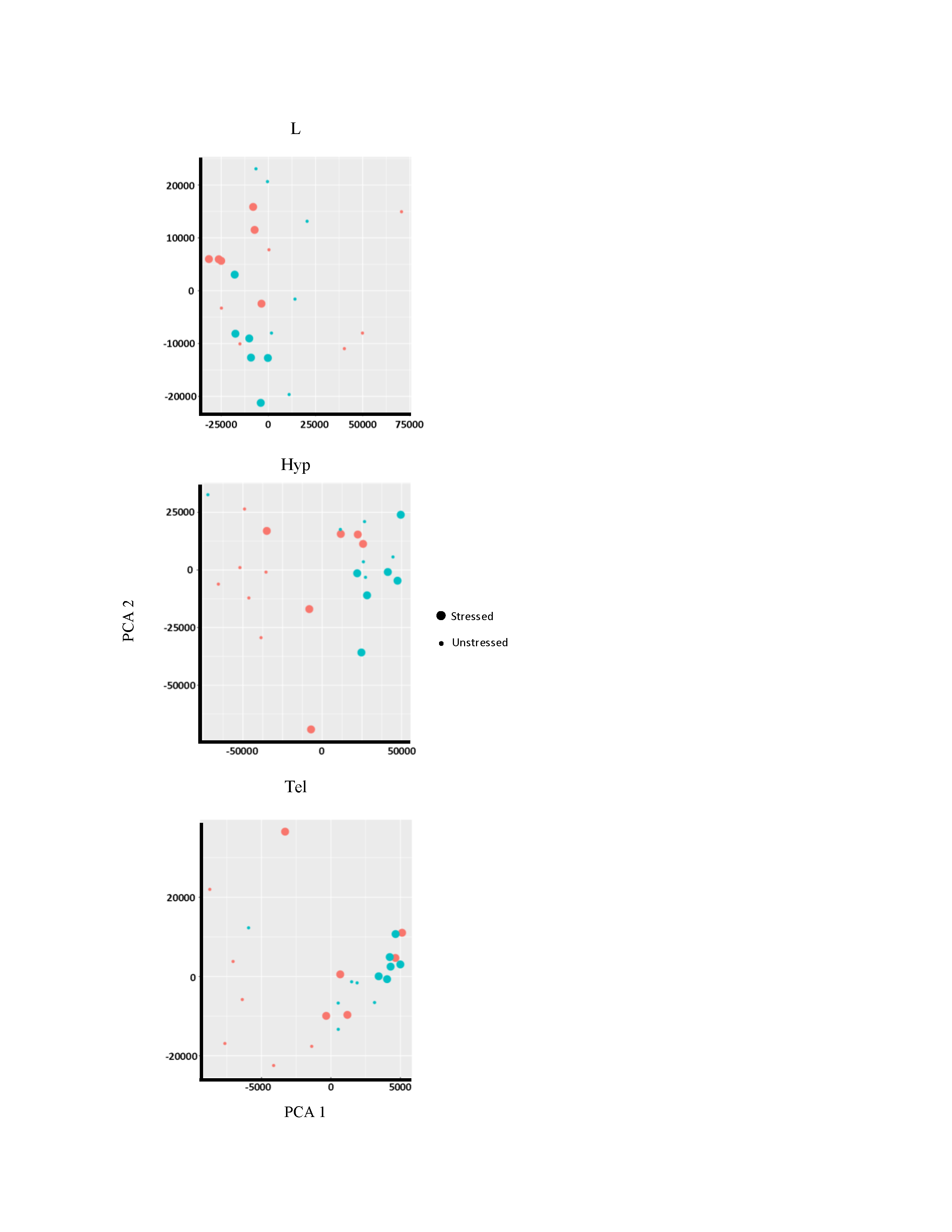

Supplement: Supplementary Figure 3 — Principle component analysis of the mapped transcriptome in fluoxetine-exposed groups compared to the controls in unstressed and stressed conditions. The PCA 1 (x-axis) and PCA 2 (y-axis) in the three studied tissues (L, Hyp, and Tel) in the unstressed and stressed conditions are shown. Only genes with FC≠≥ 1.2 are depicted here. The blue circles represent the FLX exposed groups, and the red circles represent the control groups. The size of the circles defines the stress status. [file Image_3.tif]

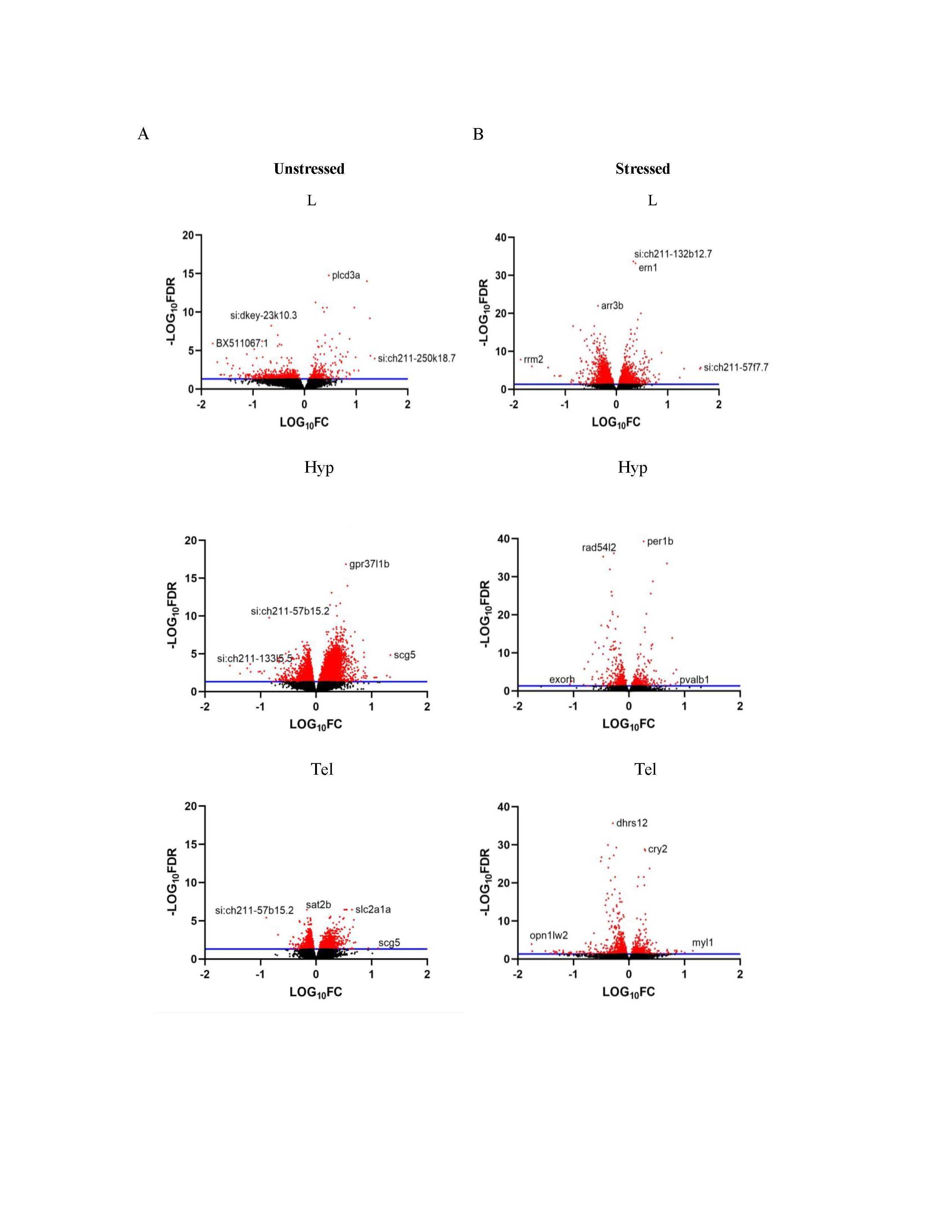

Supplement: Supplementary Figure 4 — Volcano plots of the mapped transcriptome in fluoxetine-exposed groups compared to the controls in unstressed and stressed conditions. The volcano plots in the three studied tissues in the unstressed and stressed conditions are shown. The x-axis shows the fold change (FC) on a logarithmic scale. Only genes with FC≥ 1.2 are depicted here. The y-axis shows the level of significance on a negative logarithmic scale. The scales of the y-axis are different for unstressed and stressed conditions to illustrate the data points clearly. The blue line shows the adjusted p-value ≤ 0.05 (equal to 1.3 on a negative logarithmic scale). The genes with significant FC are demonstrated as red dots above the blue threshold line. The genes with the most significant fold change and the highest significance are labeled in each graph. L, larval heads; Hyp, hypothalamus; Tel, telencephalon. [file Image_4.tif]
